# Supplementary material for: Association between Global Monkeypox Cases and Meteorological Factors
Source: Int J Environ Res Public Health. 2022 Nov 24;19(23):15638. doi: 10.3390/ijerph192315638 (PMC9740470; doi:10.3390/ijerph192315638)
Supplement: Supplementary file 1 [file ijerph-19-15638-s001.zip › Supplementary file.pdf]

Table S1. Descriptive statics of daily Monkeypox confirmed case and metrological parameter in the world during June to September 2022 by six continents.

Continent Descriptive

Temperature (°C)						
	**Africa**	**Asia**	**Europe**	**North America**	**Oceania**	**South America**
**Minimum**	4.54	6.73	-4.81	3.04	6.77	1.29
**Maximum**	33.8	39.58	32.62	32.51	21.16	28.44
**Mean ± SD**	23.7 ± 5.7	26.55±6.88	16.98±6.03	23.95±4.98	14.53±4.06	19.45±8.72

Dew/frost point temperature(°C)						
	**Africa**	**Asia**	**Europe**	**North America**	**Oceania**	**South America**
**Minimum**	-8.7	-8.26	-5.91	3.04	3.39	-7.20
**Maximum**	23.8	27.26	25.2	32.51	25.04	25.66
**Mean ± SD**	16.7±7.6	14.77±7.46	10.90± 4.36	23.95±4.98	15.81± 7.25	14.91±9.36

Relative humidity (%)						
	**Africa**	**Asia**	**Europe**	**North America**	**Oceania**	**South America**
**Minimum**	12.5	6.38	17.19	19.44	53.12	36.50
**Maximum**	97.8	94.44	98.12	97.44	95.44	97.25
**Mean ± SD**	70.3± 17.6	58.38±23.39	70.68± 14.70	78.09±14.79	80.59± 8.30	78.29±14.86

Precipitation (mm/day)						
	**Africa**	**Asia**	**Europe**	**North America**	**Oceania**	**South America**
**Minimum**	0.00	0.00	0.00	0.00	0.00	0.00
**Maximum**	157.00	137.03	66.73	308.09	64.81	173.65
**Mean ± SD**	4.6± 10.6	3.96±10.98	2.44± 5.90	9.14±24.88	3.08± 8.05	6.67±15.53

Surface pressure (kPa)						
	**Africa**	**Asia**	**Europe**	**North America**	**Oceania**	**South America**
**Minimum**	88.6	86.67	87.22	79.50	96.50	83.80
**Maximum**	99.6	101.18	102.93	102.59	103.29	102.37
**Mean ± SD**	94.3± 3.5	95.30±4.62	96.83± 3.72	96.37±6.36	100.74± 1.66	96.50±5.64

Wind speed (m/s)						
	**Africa**	**Asia**	**Europe**	**North America**	**Oceania**	**South America**
**Minimum**	0.2	0.51	0	0.16	1.57	0.11
**Maximum**	6.1	9.08	9.27	10.91	10.45	4.60
**Mean ± SD**	1.8± 0.9	2.55±1.31	1.93± 1.31	2.98±2.12	5.08± 1.93	0.87±0.95

Daily confirmed case (number of person)						
	**Africa**	**Asia**	**Europe**	**North America**	**Oceania**	**South America**
**Minimum**	0.00	0.00	0.00	0.00	0.00	0.00

**Maximum**	130.0	25.00	560.00	1392	18.00	411.00
**Mean ± SD**	0.63± 6.04	0.16±1.13	4.21± 26.38	17.96±97.13	0.47 ± 1.87	13.99±47.26

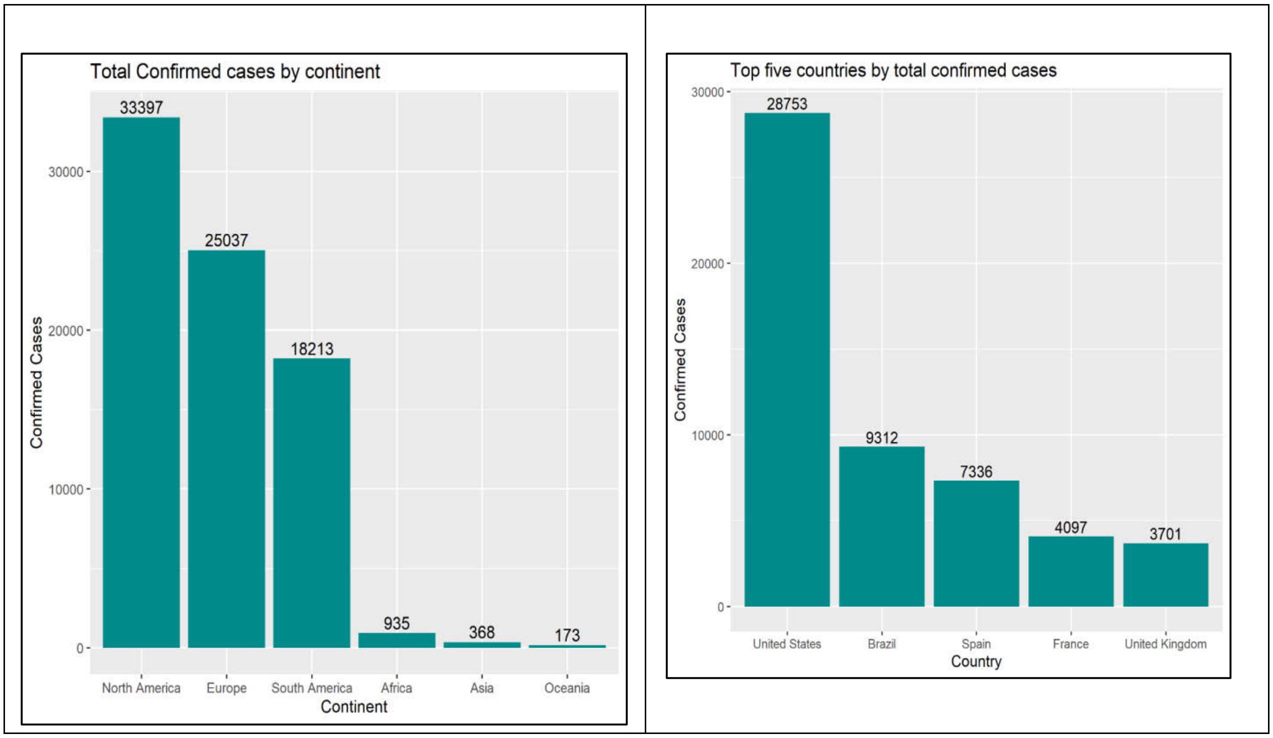

Figure S1. Top five countries based in continent and total confirmed case.
